# The DNA helicase RTEL1 is involved in the repair of replicative DNA damage independently of the alternative end joining and the DNA–protein cross‐link repair pathways in *Arabidopsis*

**DOI:** 10.1111/tpj.70903

**Published:** 2026-05-05

**Authors:** Lara Goldkuhle, Laura Lechner, Niklas Capdeville, Annika Dorn, Holger Puchta

**Affiliations:** ^1^ Joseph Gottlieb Kölreuter Institute of Plant Sciences (JKIP), Molecular Biology, Karlsruhe Institute of Technology Fritz‐Haber‐Weg 4 Karlsruhe 76131 Germany

**Keywords:** *Arabidopsis thaliana*, RTEL1, replicative DNA repair, POLQ, alternative non‐homologous end joining (aNHEJ), WSS1A, DNA–protein cross‐link (DPC) repair

## Abstract

Recent studies indicate that the Fe–S DNA helicase RTEL1 plays an important role in replicative DNA repair in plants. Since non‐homologous end joining (NHEJ) and DNA–protein cross‐link (DPC) repair also protect DNA replication in *Arabidopsis*, we set out to further define the interactions of RTEL1 and these pathways by analyzing double mutants. Plants with simultaneous defects in the alternative NHEJ factor POLQ and RTEL1 were severely restricted in growth and fertility. While both single mutants showed mitotic anaphase chromosome fragmentation at low frequencies, the simultaneous loss of both factors aggravated the effect by an order of magnitude. In contrast, simultaneous knockout of the classical NHEJ factor KU70 and RTEL1 hardly hampered proliferation and did not lead to any enhancement in chromosome fragmentation. This finding indicates that RTEL1 acts in a parallel pathway to the alternative NHEJ but not classical NHEJ in repairing aberrant replication intermediates. Simultaneous loss of RTEL1 and the protease WSS1A, which is involved in DPC repair, also showed strongly enhanced growth defects, enhanced sensitivity to cross‐linking agents and chromosomal aberrations. Thus, RTEL1 is also able to handle aberrant proteinoid DNA replication intermediates but independently of DPC repair.

## Significance Statement

Safeguarding DNA replication requires coordinated action of multiple repair pathways, yet how these factors cooperate remains unclear. By characterizing double mutants, we could now demonstrate that the DNA helicase RTEL1 acts independently of DNA–protein cross‐link repair and the alternative pathway of non‐homologous end joining (NHEJ) but in concert with the classical NHEJ.

## INTRODUCTION

A row of different DNA repair pathways safeguard proper DNA replication in plants. Both genomic double‐strand breaks (DSBs) and DNA–protein adducts are severe obstacles for the replication machinery. *Arabidopsis* mutants defective in key components of DNA repair pathways for both kinds of damages exhibit growth retardation (Enderle et al., 2019; Inagaki et al., 2006; Nisa et al., 2021) as well as chromosomal breakage and aberrations during mitotic anaphase (Hacker et al., 2022; Merker et al., 2024).

In somatic plant cells, most DSBs are repaired by non‐homologous end joining (NHEJ). Two pathways, defined as classical (c) and alternative (a) or microhomology‐mediated NHEJ, operate in this process (Puchta, 2005). The KU70/80 heterodimer, together with Ligase 4, plays a central role in cNHEJ (Bundock et al., 2002; Tamura et al., 2002), whereas POLQ, also called TEBICHI, is the key factor for aNHEJ (Inagaki et al., 2006; van Kregten et al., 2016; van Schendel et al., 2015). While KU70 deletion mutants exhibit only minor growth defects, depending on growth conditions, severe growth retardation has been reported for mutants of POLQ (Nisa et al., 2021), indicating that aNHEJ is especially important for safeguarding DNA replication. Previously, we generated *ku70‐1/teb‐5* double mutants. As these showed massive growth retardation and also—in contrast to the single mutants—high rates of somatic chromosome breakage, both NHEJ pathways can complement each other in DSB repair to a considerable extent (Merker et al., 2024).

A central factor involved in the removal of DPCs is the DNA‐dependent protease WSS1A. Arabidopsis mutants lacking WSS1A display mild growth retardation (Enderle et al., 2019) and increased chromosomal aberrations (Hacker et al., 2022), indicating an important role for WSS1A in replicative DNA repair. This is further supported by the observation that these mutants show a significant reduction in 45S rDNA tandem repeat number, which is a characteristic feature of Arabidopsis mutants defective in replicative DNA repair (Dorn et al., 2019; Röhrig et al., 2016). To determine whether WSS1A functions in concert with either of the two NHEJ pathways, the respective double mutants were generated by crossing and analyzed. Both double mutants exhibited aggravated growth defects. However, the loss of aNHEJ resulted in a much stronger growth defect than the loss of cNHEJ. In addition, a massive increase in chromosome fragmentation was only detected in the *teb‐5/wss1a‐3* double mutant, indicating that POLQ and WSS1A are both important factors safeguarding DNA replication in independent pathways (Hacker et al., 2022).

It has been shown that the helicase RTEL1 fulfills multiple roles in safeguarding DNA replication in eukaryotes by facilitating telomere replication, resolving R‐loops, D‐loops, G‐quadruplex structures, as well as by preventing transcription–replication collisions (Hourvitz et al., 2024; León‐Ortiz et al., 2018). In Arabidopsis, RTEL1 has been shown to be involved in replicative DNA repair (Hu et al., 2015), DNA cross‐link repair (Recker et al., 2014), and transcriptional silencing (Olivier et al., 2023). Loss of RTEL1 also results in a significant reduction of 45S rDNA tandem repeats in the Arabidopsis genome (Dorn et al., 2019; Röhrig et al., 2016).

The aim of the current study was to determine whether RTEL1 acts together with the end joining pathways or with WSS1A in DNA repair. Alternatively, it might also be capable of resolving replication‐blocking lesions or DNA repair intermediates largely independently of these factors.

## RESULTS

### Establishment of a AtPOLQ/AtRTEL1 double mutant

**Figure 1:**
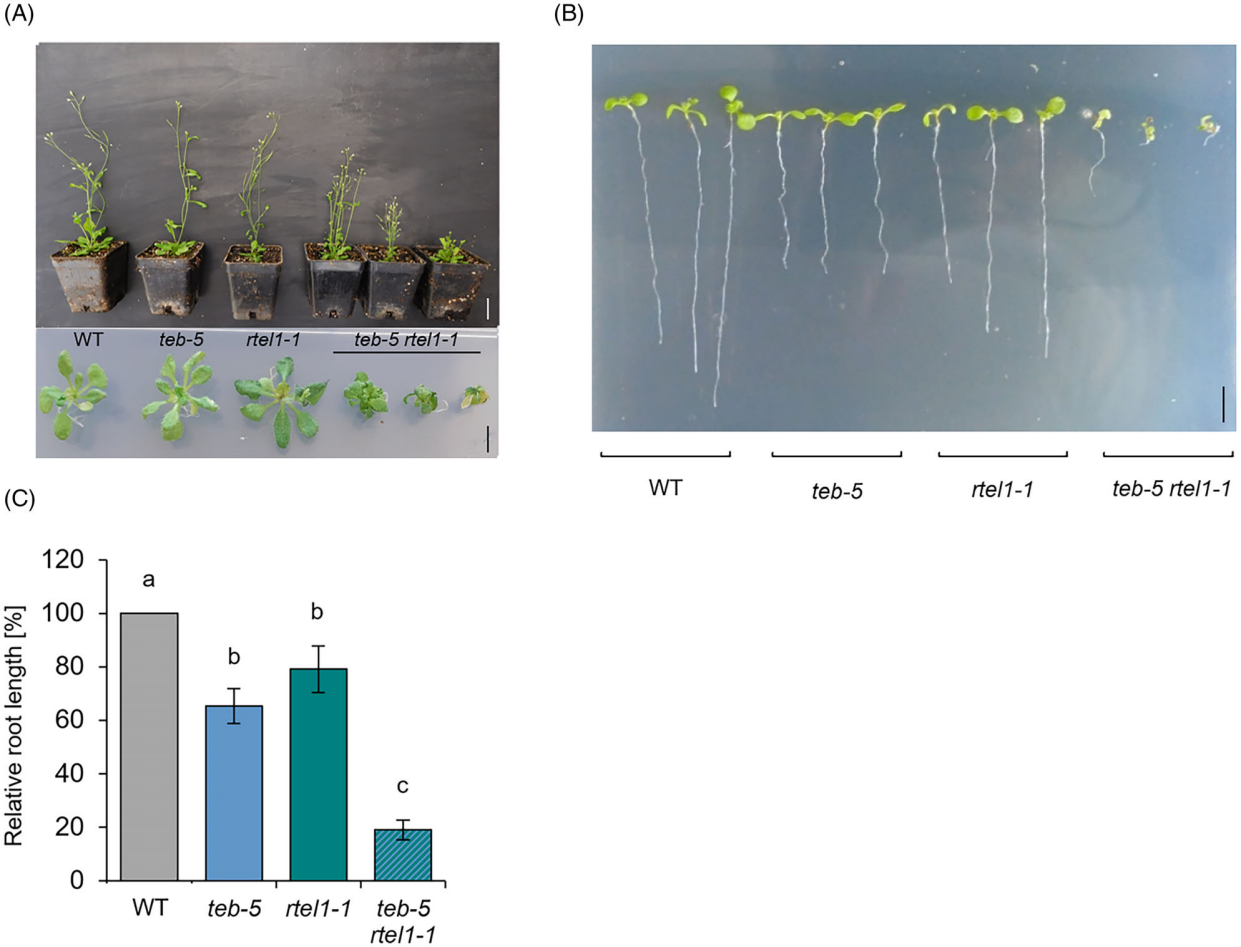
Growth phenotype of *teb‐5/rtel1‐1*. (A) Shown are 5‐week‐old (upper figure) and 3‐week‐old seedlings (lower figure) of *teb‐5/rtel1‐1* double mutants compared with wild‐type (WT) plants and the respective single mutants, all in the Columbia‐0 background. The double mutants exhibit severe growth impairment (upper scale bar = 2 cm, lower scale bar = 0.5 cm). (B, C) Root length in 10‐day‐old *teb‐5/rtel1‐1* seedlings is significantly reduced compared with wild‐type plants and the respective single mutants (scale bar = 0.5 cm, *n* = 3). Statistical differences were calculated using one‐way ANOVA followed by Tukey's post hoc test and are presented as *a* ≠ *b* when *P* < 0.05.

In a recent study, it has been reported that crossing the *rtel1‐1* mutant with the POLQ mutant *teb‐1* did not result in viable progeny (Dvořák Tomaštíková et al., 2024). However, as the phenotype of POLQ mutants is highly variable depending on growth conditions (Nisa et al., 2021), we attempted to generate the double mutant by crossing *rtel1‐1* with the originally described *teb‐5* mutant (Inagaki et al., 2006). Indeed, under our growth conditions, we obtained viable seeds, allowing us to characterize the phenotypic and molecular consequences of the simultaneous loss of both pathways. A conspicuous small growth phenotype was observed with the double mutant compared with wild‐type plants and the corresponding single mutants (Figure 1A). The plants also exhibited deformed, poorly developed leaf morphology. Root length analyses were performed to quantify these growth deficits. Because the root meristem is a rapidly dividing tissue, it is an optimal system for analyzing replicative DNA damage (Curtis & Hays, 2007). Lesions that occur spontaneously during replication impair cell division and therefore directly correlate with root growth. For this analysis, the primary root length of 9‐day‐old seedlings of the double mutant, the two corresponding single mutants and wild‐type plants was measured using ImageJ in combination with the SmartRoot plugin. To analyze a potential genetic interaction between AtPOLQ and AtRTEL1 in the repair of replication‐dependent DNA damage and to further quantify the impaired growth of the double mutant, the primary root length of *teb‐5/rtel1‐1* was compared with wild‐type plants as well as the corresponding single mutants. Indeed, the roots of the double mutant were barely developed and visibly shortened (Figure 1B). A detailed evaluation showed an average reduction in root length of nearly 80% in AtPOLQ/AtRTEL1‐deficient plants, compared with wild‐type plants (Figure 1C). Significantly reduced root length of the double mutant was also observed in comparison with *teb‐5 and rtel1‐1* single mutants. In summary, the combined loss of AtPOLQ and AtRTEL1 strongly compromises root growth. This suggests that both proteins contribute to processes essential for proper root development in parallel pathways, and potentially play a role in the repair of replication‐associated DNA damage.

### Combined loss of AtRTEL1 and AtPOLQ results in sterility mainly due to pre‐meiotic defects

The ability to grow seedlings of the double mutant *teb‐5/rtel1‐1* allowed us to characterize the resulting defects in greater detail. When examining the fertility of the double mutant, we unexpectedly observed numerous seedless siliques. Analysis of at least five siliques from five individual plants per line revealed an average of 50 seeds per silique in wild‐type plants. Both *teb‐5* and *rtel1‐1* single mutants displayed comparable fertility. In contrast, siliques of the *teb‐5/rtel1‐1* double mutant contained only four seeds on average (Figure S1A). Thus, the combined loss of AtPOLQ and AtRTEL1 results in even more drastically reduced fertility than was previously discovered for a simultaneous mutation in AtPOLQ and AtKU70 (Merker et al., 2024).

To uncover the cause of this dramatically impaired fertility in the *teb‐5/rtel1‐1* double mutant, we examined pollen viability using Alexander staining. It has already been shown that the loss of AtRTEL1 leads to a significantly reduced pollen number compared with wild‐type plants (Röhrig et al., 2016). In our analysis, no dead pollen grains were detected in any of the tested genotypes (Figure S1B). However, all anthers of *teb‐5/rtel1‐1* were severely deformed. The amount of pollen was also drastically reduced and often only a single pollen sac contained pollen grains, while some anthers were completely devoid of pollen. These results indicate that both AtPOLQ and AtRTEL1 are essential for proper male gametogenesis and that their simultaneous loss severely compromises gamete development.

To determine whether the impaired fertility of the *teb‐5/rtel1‐1* double mutant is caused solely by defects in male gametogenesis or whether the female germline is also affected, we performed reciprocal backcrosses with wild‐type plants. The double mutant was used once as the female and once as the male crossing partner. As a control, a crossing between two wild‐type plants was also performed. The siliques of plants backcrossed with *teb‐5/rtel1‐1* as the crossing partner contained significantly fewer seeds per silique compared with wild‐type controls, and both reciprocal combinations produced comparable results (Figure S2). This indicates a gender‐unspecific fertility defect. Both male and female gametogenesis appear to be affected by the simultaneous loss of AtPOLQ and AtRTEL1.

**Figure 2:**
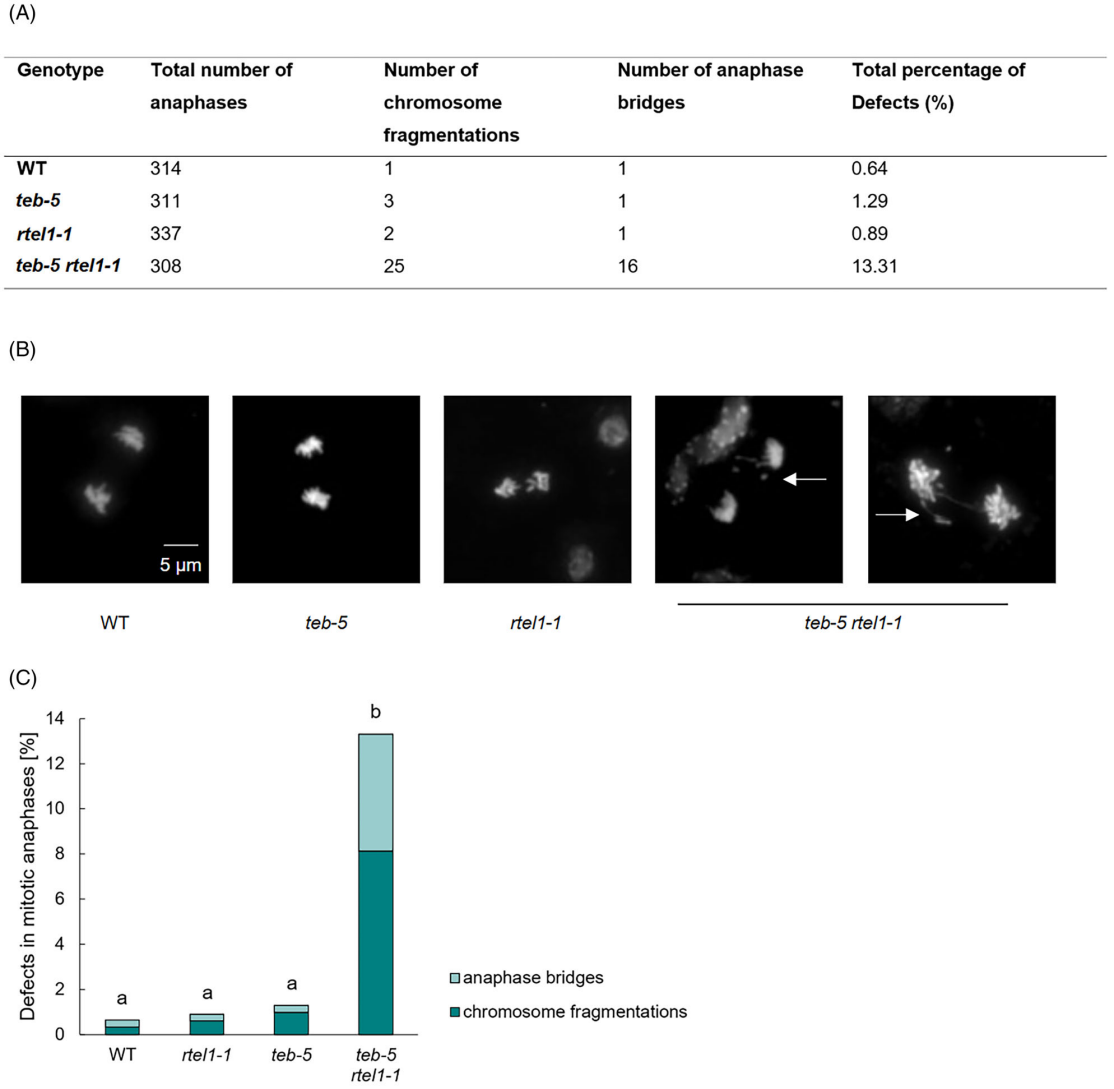
Mitotic anaphases in *teb‐5/rtel1‐1*. (A) Shown is the number of detected defects (chromosome fragmentations and anaphase bridges) within the mitotic anaphases of *teb‐5/rtel1‐1* in relation to both single mutants and the wild‐type (WT). (B, C) DAPI‐stained chromosome spreads were analyzed to identify defects in mitotic anaphases. For each genotype, at least 300 mitotic anaphases derived from a minimum of three independent plants were analyzed. For *teb‐5/rtel1‐1*, a significant increase in anaphase bridges and chromosome fragmentations could be detected in comparison with the wild‐type and the corresponding single mutants. Statistical differences were calculated using a two‐tailed Fisher's exact test and are presented as *a* ≠ *b* when *P* < 0.05.

To further investigate the cause of the fertility defect, male meiocytes were microscopically analyzed. As meiotic progression appeared unaffected, the defect is likely to originate at an earlier stage. Following DAPI staining of chromatin preparations from inflorescences, mitotic anaphases could be visualized (Figure 2A–C). The *rtel1‐1* single mutant did not show any abnormalities and only approximately 1.3% of AtPOLQ‐deficient cells displayed defects. However, the combined loss of AtPOLQ and AtRTEL1 resulted in substantially greater damage during mitotic anaphases. In approximately 13% of *teb‐5/rtel1‐1* nuclei, anaphase progression was defective. Thereby, chromosome fragments and anaphase bridges of incompletely separated chromosomes could be detected. Consequently, the fertility defect observed in *teb‐5/rtel1‐1* mutants can be attributed mainly to pre‐meiotic defects.

### Simultaneous loss of AtKU70 and AtRTEL1 does not lead to a major growth defect

**Figure 3:**
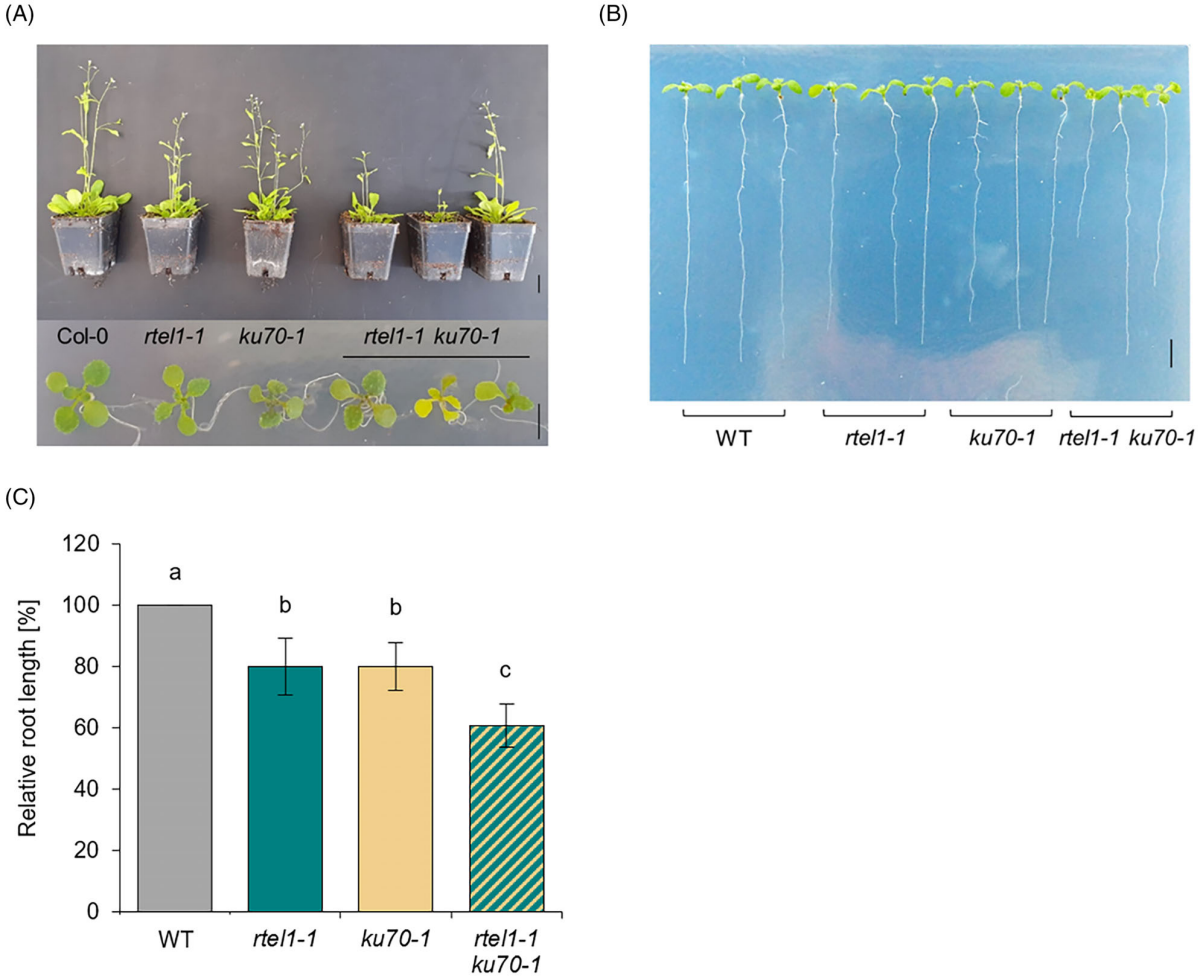
Growth phenotype of *rtel1‐1/ku70‐1*. (A) Shown are 5‐week‐old (upper figure) and 3‐week‐old seedlings (lower figure) of *rtel1‐1/ku70‐1* double mutants compared with wild‐type (WT) plants and the respective single mutants, all in the Columbia‐0 background. The double mutants exhibit a slight growth impairment (upper scale bar = 2 cm, lower scale bar = 0.5 cm). (B, C) Root length in 10‐day‐old *rtel1‐1/ku70‐1* seedlings is significantly reduced compared with wild‐type plants and the respective single mutants (scale bar = 0.5 cm, *n* = 3). Statistical differences were calculated using a one‐way ANOVA followed by Tukey's post hoc test and are presented as *a* ≠ *b* when *P* < 0.05.

As the simultaneous loss of AtPOLQ in combination with either AtKU70 or AtRTEL1 causes severe growth retardation, indicating major defects in the repair of replicative DNA damage, we next examined whether the At*ku70/*At*rtel1* double mutant was similarly impaired in proliferation. Interestingly, growth defects in this mutant were much less severe than those observed in *teb‐5/ku70‐1* or *teb‐5/rtel1‐1* double mutants and root length was only slightly reduced (Figure 3). Additional analysis of the average seed number per silique showed that the combined loss of AtRTEL1 and AtKU70 had only a very mild effect on fertility. This indicates that, in contrast to AtPOLQ, AtKU70 and AtRTEL1 primarily function either in the same pathway or in dependent processes.

As we observed a massive increase in chromosomal aberrations in the *rtel1‐1* mutant in the absence of the aNHEJ, we also tested whether loss of cNHEJ had a similar effect. Although, as expected, we were able to detect a slight increase in aberrations in the *ku70‐1* mutant, similar to the *rtel1‐1* mutant, we did not find any further enhancement in the double mutant (Figure S3). These findings further support the idea that both factors act either in the same pathway or in dependent processes. It is tempting to speculate that RTEL1 may resolve replication‐associated obstacles into DSBs, which are subsequently processed by the cNHEJ pathway.

### RTEL1 and WSS1A act independently in replicative DNA repair

The DNA‐dependent protease WSS1A is required for proper replication, as it removes stalled topoisomerase–DNA intermediates and other kinds of proteinaceous DNA adducts. Previous studies demonstrated that SMC5/SMC6, which are involved in a WSS1A‐independent pathway of DPC repair (Dvořák Tomaštíková et al., 2023), act independently of RTEL1 (Dvořák Tomaštíková et al., 2024). It was therefore interesting to investigate whether WSS1A and RTEL1 are participating in a common pathway.

**Figure 4:**
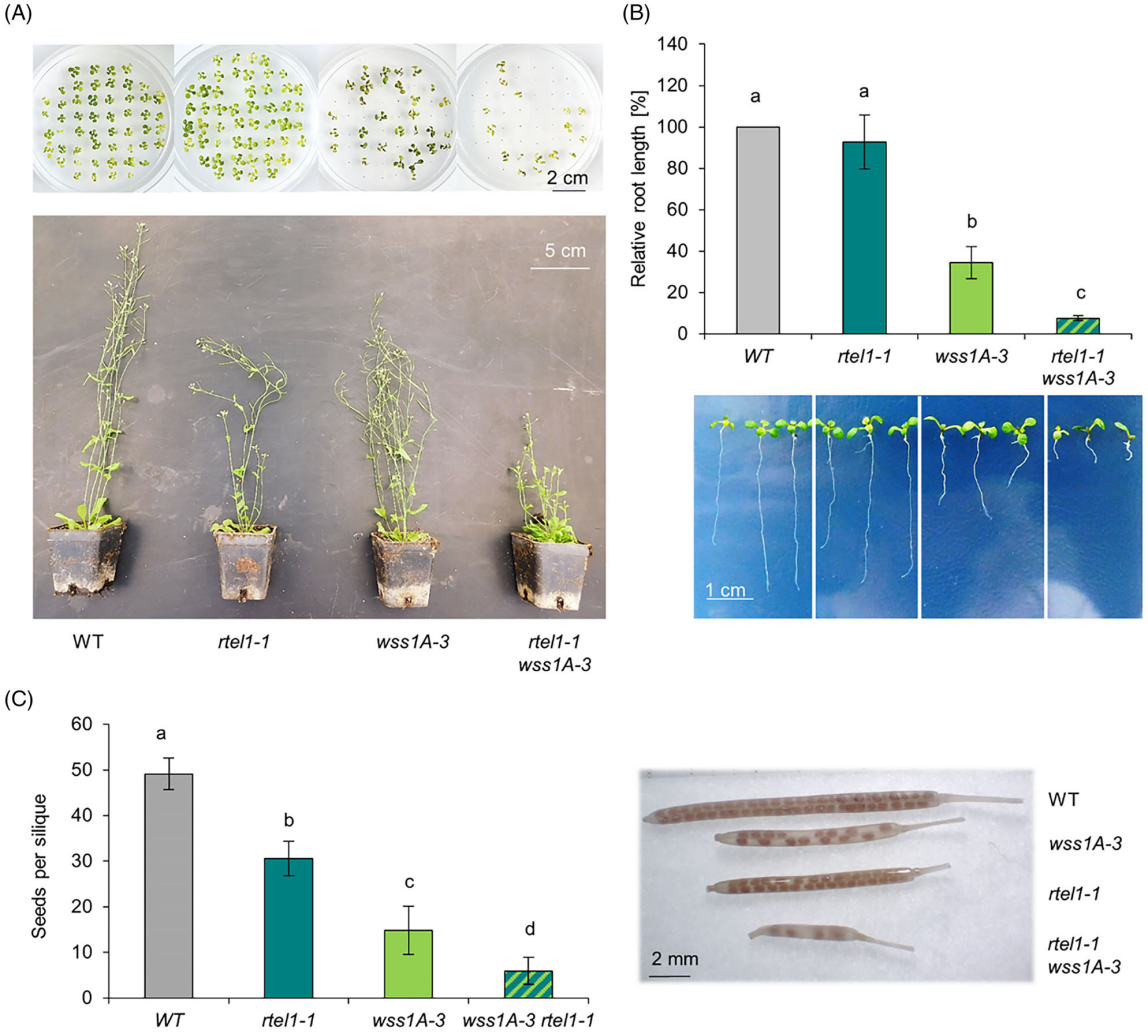
Epistasis analysis of WSS1A and RTEL1 in growth, fertility. (A) Shown are 5‐week‐old (upper figure) und 3‐week‐old seedlings (lower figure) of *rtel1‐1/wss1a‐3* double mutants compared with wild‐type (WT) plants and the respective single mutants, all in the Columbia‐0 background. Analysis of the double mutants revealed strong developmental defects. (B) Root length in 10‐day‐old *rtel1‐1/wss1a‐3* seedlings is significantly reduced compared with wild‐type plants and the respective single mutants (*n* = 3). (C) Quantification of the average number of seeds per silique showed a significantly reduced fertility of *rtel1‐1/wss1A‐3* compared with the wild‐type and the respective single mutants (*n* = 5). Statistical differences were calculated using a one‐way ANOVA followed by Tukey's post hoc test and are presented as *a* ≠ *b* when *P* < 0.05.

Interestingly, we were able to obtain viable plants which exhibited a severely restricted growth phenotype (Figure 4A). Root length analyses were performed to quantify the observed growth deficits. For the analyses, root length of 9‐day‐old seedlings of the double mutant as well as the two corresponding single mutants and wild‐type plants was measured using ImageJ in combination with the SmartRoot plugin. While *rtel1‐1* roots were comparable to the roots of wild‐type plants, *wss1A‐3* mutants displayed a significantly reduced root length (Figure 4B). The simultaneous loss of both factors, on the other hand, resulted in a significant reduction in root growth compared with either single mutant. In comparison to the wild‐type, *rtel1‐1 wss1A‐3* double mutants showed a decrease in root length of up to 92.5%. These results provide strong indirect evidence that RTEL1 and WSS1A act in parallel pathways influencing processes associated with replication‐dependent DNA repair.

Since it has already been shown that both RTEL1 and WSS1A contribute to proper fertility, we were interested in analyzing the fertility of the double mutant *rtel1‐1/wss1A‐3* (Röhrig et al., 2016). To this end, we determined the average number of seeds per silique. For both single mutants, a significantly reduced seed number of 31 (*rtel1‐1*) and 15 (*wss1A‐3*), respectively, was detected in relation to the wild‐type (49 seeds). For *rtel1‐1/wss1A‐3*, even further reduction in fertility was observed with an average of 6 seeds per silique (Figure 4C). The obtained results indicate that both factors contribute to cell proliferation in somatic tissues and partly complement each other.

**Figure 5:**
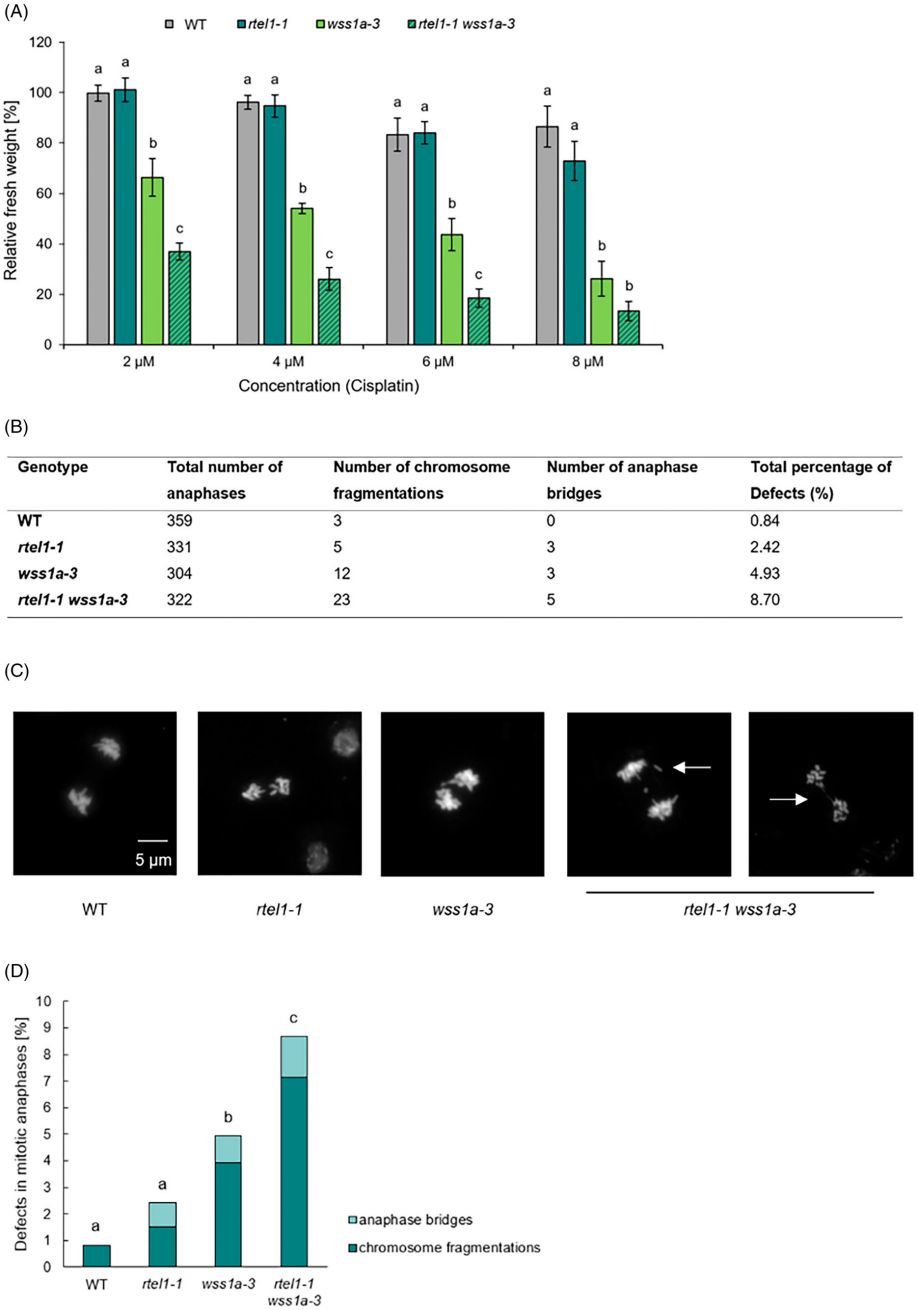
Sensitivity analysis and mitotic anaphases of *rtel1‐1/wss1a‐3*. (A) The fresh weight of *rtel1‐1/wss1a‐3* double mutants, wild‐type (WT) plants and the corresponding single mutants was measured after 13 days exposure to cisplatin. Relative fresh weight values were calculated using untreated control samples of each genotype. Shown is the mean relative fresh weight from at least three independent assays. After treatment with 2, 4, 6, and 8 μM cisplatin, *wss1A‐3* showed a significant reduction in relative fresh weight (66–26%) compared with the wild‐type, while the double mutant *rtel1‐1/wss1A‐3* exhibited an even greater decrease (37–13%). Statistical differences were calculated using a one‐way ANOVA followed by Tukey's post hoc test and are presented as *a* ≠ *b* when *P* < 0.05. (B) Shown is the number of detected defects, chromosome fragmentations and anaphase bridges during mitotic anaphases in *rtel1‐1/wss1a‐3*, compared with the respective single mutants and the wild‐type. (C, D) DAPI‐stained chromosome spreads were analyzed to identify defects in mitotic anaphases. For each genotype, at least 300 mitotic anaphases derived from a minimum of three independent plants were analyzed. For *rtel1‐1/wss1a‐3*, a significant increase in anaphase bridges and chromosome fragmentations could be detected in comparison to the wild‐type and the corresponding single mutants. Statistical differences were calculated using a two‐tailed Fisher's exact test and are presented as *a* ≠ *b* when *P* < 0.05.

To further investigate the role of RTEL1 and WSS1A in the repair of aberrant replication intermediates, we analyzed the sensitivity of the *rtel1‐1/wss1a‐3* double mutant to the cross‐linking agent cisplatin (Figure 5A). Following treatment with 2, 4, 6, and 8 μM cisplatin, the relative fresh weight of the *wss1a‐3* single mutant was significantly reduced to 66–26% compared with the wild‐type (100–86%). In addition, the *rtel1‐1/wss1a‐3* double mutant exhibited an even stronger reduction, ranging from 37 to 13%.

As we observed a strong increase in chromosomal aberrations in the *rtel1‐1* mutant in the absence of the aNHEJ factor POLQ, but not in the absence of the cNHEJ factor KU70, we next investigated how the loss of the DPC repair factor WSS1A would affect mitotic progression. To this end, chromatin preparations from inflorescences were stained with DAPI to visualize mitotic anaphases (Figure 5B–D). The *rtel1‐1* single mutant exhibited 2.4% defective mitotic anaphases, including chromosome fragmentations and anaphase bridges. The *wss1A‐3* single mutant showed 4.9% defects. In the *rtel1‐1/wss1A‐3* double mutant, chromosomal aberrations were significantly enhanced to 8.7%. Thus, all data obtained with the double mutant showed an additive to synergistic increase in the respective deficiencies detected in the single mutants, demonstrating that AtRTEL1 and AtWSS1A act predominantly in independent pathways of DPC repair.

## DISCUSSION

A range of distinct DNA repair pathways safeguards the genome against DNA damage arising during DNA replication. Many of these pathways are conserved among eukaryotes. However, the relative contribution of these individual pathways might differ drastically between animals, yeast, and plants.

If factors with an essential role in replicative DNA repair are deficient in Arabidopsis, the respective plants exhibit growth retardation. Frequently, other pathways are able to partially compensate for this defect, either by removing replication obstacles or by assisting in the repair of the resulting damage. However, when factors of these compensatory pathways are also lost, much more severe growth deficiencies occur, in some cases resulting in complete lethality.

We previously demonstrated that the simultaneous removal of the key factors involved in both alternative (POLQ) and classical (KU70) end joining results in severe growth defects (Merker et al., 2024). Similarly, double mutants of POLQ and the DPC repair protease WSS1A exhibit pronounced growth retardation (Hacker et al., 2022). Moreover, double mutants of WSS1A and the DNA helicase and RTR complex partner RECQ4A resulted in embryo lethality (Hacker et al., 2022). Interestingly, RECQ4A deficiency in combination with the helicase RTEL1 also leads to severe growth defects (Recker et al., 2014).

In contrast to the RTR complex, NHEJ or DPC repair, the roles of RTEL1 in DNA repair in Arabidopsis are less clearly defined, particularly with respect to its interactions with other repair pathways. Previous studies have shown that AtRTEL1 is involved in telomere homeostasis (Olivier et al., 2018), suppression of intrachromosomal homologous recombination, DNA cross‐link repair (Recker et al., 2014), replicative DNA repair (Hu et al., 2015), DPC repair (Dvořák Tomaštíková et al., 2024), 45S rDNA stability (Röhrig et al., 2016), as well as transcriptional silencing and epigenome stability (Olivier et al., 2023).

But what unique biochemical properties does RTEL1 possess to process DNA repair and replication intermediates? From animal cells we know that RTEL1 can dissolve D‐loops that arise during homologous recombination (Barber et al., 2008), R‐Loops generated during transcription (Björkman et al., 2020), and T‐Loops at telomeres (Sarek et al., 2015) through its helicase activity. Moreover, it can resolve G4 structures and prevent replication–transcription collisions (Kotsantis et al., 2020). Thus, two scenarios can be envisaged in which the helicase activity of RTEL1 is relevant for suppression of instabilities during DNA replication: On the one hand, by removing replication obstacles such as G4 structures or R‐Loops, whose presence could block DNA replication or lead to DSB formation. On the other hand, RTEL1 might also be directly involved in DNA repair reactions by dissolving D‐loops during homologous recombination reactions and channeling them into other repair pathways. Indeed, studies in animal cells strongly indicate that RTEL1 is likely involved in both types of processes (Hourvitz et al., 2024).

RTEL1 does not appear to play a predominant role in either of the two protease‐based branches of DPC repair pathways in Arabidopsis, as double mutants with the protease WSS1A (this work) or SMC6b, a component of the proteasome‐based degradation‐dependent pathway (Dvořák Tomaštíková et al., 2023), both exhibit growth deficiencies and reduced fertility. In addition to the severe reduction in fertility observed in the *wss1a‐3/rtel1‐1* double mutant, we observed a reduction in root length growth by 90% (Figure 4) compared with the 50% reduction reported for the *smc6b‐1*/*rtel1‐1* double mutant.

This indicates that RTEL1 might be involved in processing DNA intermediates that arise from alternative pathways of DPC repair. The nuclease MUS81 acts in a parallel pathway to SMC5/6 and WSS1A in resolving DPCs through nucleic acid degradation. The resulting intermediates could then be processed by RTEL1. In line with this hypothesis is the fact that we previously showed that RTEL1 and MUS81 act in a common pathway repairing interstrand DNA cross‐links in Arabidopsis (Recker et al., 2014). The observation that we detected more chromosomal breaks in the mitotic anaphases of the *rtel1‐1 wss1a‐3* double mutant (Figure 5) is consistent with the idea that processing of DNA intermediates by RTEL1 may help to prevent persistent DSB formation.

In somatic plant cells, DSBs are repaired by two NHEJ pathways that can at least partially compensate for each other, as the respective double mutant shows massive growth retardation (Merker et al., 2024). Surprisingly, the phenotypes of the *rtel1‐1* double mutants with both NHEJ pathways differed drastically. The double mutant with POLQ displayed, in our hands, severe growth retardation and a pre‐meiotic fertility defect (Figure 1), while others even reported lethality under their growth conditions (Dvořák Tomaštíková et al., 2024). The fact that we were able to obtain viable plants enabled us to characterize the resulting repair defects in more detail. Indeed, we detected a strong increase in chromosomal aberrations when examining mitotic anaphases. Interestingly, both chromosome fragmentations and anaphase bridges were massively enhanced. This pattern provides strong indirect evidence that, in the absence of RTEL1, replicative DNA repair intermediates may persist and give rise to DSBs that would normally be repaired by aNHEJ. Moreover, some of these breaks might instead be religated by cNHEJ, potentially in an incorrect manner, thereby linking different chromosomes and giving rise to anaphase bridges.

Although we detected a massive reduction of fertility in the *teb‐5/rtel1‐1* double mutant, we observed no major disturbance of meiosis in these plants. It should be noted that the double mutant exhibited severe developmental and growth defects. Root size was reduced by 80%, and plants were severely stunted and malformed (Figure 1). This indicates that all cell divisions in the respective meristems are strongly hampered during somatic growth. This effect may be further exaggerated during meiotic proliferation, as multiple divisions occur preceding male gametogenesis. Although pollen numbers were reduced by 90%, we did not detect non‐viable pollen. Moreover, in contrast to pre‐meiotic anaphases (Figure 2), no major defects in meiotic figures were observed. Collectively, these observations indicate that the reduction in pollen numbers is primarily due to a massive reduction of pre‐meiotic progenitor cells.

In contrast to the *teb‐5/rtel1‐1* double mutant, the *ku70‐1/rtel1‐1* double mutant showed only a mild growth retardation and not the slightest increase in chromosomal aberrations, neither in anaphase bridges nor in unrepaired DSBs. This observation is consistent with the idea that both factors act within the same repair pathway or in dependent processes. We assume that certain replication intermediates processed by RTEL1 might result in DSBs that are channeled into the cNHEJ pathway.

It has been shown that loss of RTEL1 enhances conservative somatic homologous recombination between repeats (Hu et al., 2015; Recker et al., 2014), a reaction best described by the synthesis‐dependent strand annealing model of recombination (Puchta, 2005). In contrast, RTEL1 appears to play only a minor role in repeat repair via the non‐conservative single‐strand annealing (SSA) mechanism (Dvořák Tomaštíková et al., 2024). Thus, it is tempting to speculate that RTEL1 dissolves annealed D‐loops during the SDSA reaction to stabilize DNA repeats in plant genomes. The resulting free DNA ends may then be predominantly processed by cNHEJ and only in its absence might aNHEJ take over.

It should be emphasized that our mechanistic model of RTEL1 function in *Arabidopsis* is primarily based on molecular and biochemical studies performed in other eukaryotes. Here, we provide only indirect evidence by analyzing cell proliferation, sensitivity assays, and mitotic figures. The next step will be to confirm our model by directly investigating genetic interactions in *Arabidopsis* with established replication stress regulators, such as components of the ATR‐dependent checkpoint pathway or factors involved in replication origin licensing and chromatin assembly. Such experiments will help to further delineate the mechanistic contributions of RTEL1 to replicative DNA repair in plants.

## MATERIALS AND METHODS

### Plant material and growth conditions

All *Arabidopsis* lines used in this study were of the Columbia (Col‐0) background. The T‐DNA insertion mutants *rtel1* (*rtel1‐1*, SALK_113285); *polq* (*teb‐5*, SALK_018851); *ku70* (*ku70‐1*, SALK_123114) as well as the CRISPR/Cas9‐generated *wss1A‐3* mutant have been described previously (Enderle et al., 2019; Inagaki et al., 2006; Jia et al., 2012; Recker et al., 2014). Double mutants were generated by crossbreeding homozygous T‐DNA insertion lines. Homozygosity was verified in the F2 generation by PCR‐based genotyping using wild‐type and T‐DNA‐specific primer combinations (Tables S1 and S2). Plants were cultivated either in a greenhouse on soil (1:1 mixture of Floraton 3 [Floragard, Oldenburg, Germany] and vermiculite [2–3 mm; Deutsche Vermiculite Dämmstoff, Sprockhövel, Germany]) at 22°C under long‐day conditions (16 h light/8 h dark), or on agar plates in growth chambers (CLU36L4, Percival Scientific) under stable axenic conditions (16 h light at 22°C/8 h dark at 22°C). For axenic growth, seeds were surface‐sterilized with 4% sodium hypochlorite, rinsed with water, and stratified overnight at 4°C before sowing on agar plates containing germination medium (GM; 4.9 g L^−1^ Murashige and Skoog, 10 g L^−1^ sucrose, and 7.6 g L^−1^ agar; pH 5.7).

### Root length analysis

Sterilized and stratified seeds were sown in rows on GM medium (1% agar) in square plates, which were incubated vertically in a growth chamber. After 9 days of incubation, root images were captured and analyzed using ImageJ in combination with the SmartRoot add‐on (Lobet et al., 2011). A minimum of three biological replicates was performed, and root length was measured for at least seven plants per replicate.

### Sensitivity assays

To analyze the sensitivity of the different Arabidopsis mutant lines to the genotoxin cisplatin, assays were performed as previously described (Hartung et al., 2007). Arabidopsis seeds were sterilized with 4% sodium hypochlorite, stratified overnight, and sown on agar plates containing GM medium. After 7 days, seedlings were transferred to six‐well plates containing either 4 or 5 mL of liquid GM medium (control batch without genotoxin). On the following day, 1 mL of the respective genotoxin concentrations was added. All genotoxic agents were dissolved in sterile ultrapure water. Following a 13‐day incubation period, the relative fresh weight of the plants was determined. Values of treated samples were normalized against the untreated control samples of the corresponding line. Each assay was conducted with at least three independent biological replicates.

### Fertility assays

To study the fertility of mutant lines, plants were cultivated in the greenhouse for approximately 4 weeks. Five mature siliques from at least five individual plants were decolorized overnight in 70% ethanol, and seed numbers per silique were determined using a binocular microscope.

Pollen viability was examined by Alexander staining of 4‐week‐old plants. Closed buds were fixed for 1 h in Carnoy solution (ethanol [99.5%]:chloroform:acetic acid [100%]; proportionally 6:3:1). Following fixation, buds were rinsed with deionized water, opened, and the anthers dissected and mounted on microscope slides. A staining solution (0.5 ml ethanol [95%], 50 μl Malachite green [1% in ethanol], 1.25 ml glycerol, 0.25 g chloral hydrate, 250 μl fuchsic acid [1% w/v in deionized water], 25 μl Orange G [1% w/v in deionized water] and 200 μl acetic acid in 5 ml deionized water) was applied, resulting in viable pollen staining red/purple and non‐viable pollen staining blue. After overnight incubation at 60°C, samples were evaluated using a binocular microscope and digital imaging. At least 20 anthers per line were analyzed.

### DAPI staining on mitotic chromosomes

Chromatin preparation of mitotic cells was carried out following the protocol established for male meiocytes (Armstrong et al., 2009). Anaphase bridges were quantified directly from the acquired images. For each genotype, at least 300 mitotic anaphases derived from a minimum of three independent plants were analyzed.

### Backcross experiments

Backcross experiments were conducted to determine whether impaired fertility results solely from defects in male gametogenesis or if the female germline is also affected. The mutant line was crossed with wild‐type plants, serving once as the female and once as the male partner. As a control, wild‐type plants were crossed with each other. After that, fertility assays were performed.

### Calculation of statistical significances

Differences in defective anaphases were evaluated with a two‐sided Fisher's exact test. Statistical differences in root length assays, sensitivity assays, and fertility assays were evaluated using a one‐way ANOVA followed by Tukey's post hoc test. Results were considered significant when *P* < 0.05 and differences are reported as a ≠ b.

## ACCESSION NUMBERS

Additional details and gene sequences for the proteins discussed in this study can be found at The Arabidopsis Information Resource (TAIR) using the following accession numbers: *RTEL1*, At1g79950; *POLQ*, At4g32700; *KU70*, At1g16970; *WSS1A*, At1g55915.

## AUTHOR CONTRIBUTIONS

Design of the research: LL, AD, and HP. Performance of the research: LL, LG, AD. Data analysis: LL, LG, NC, AD, and HP. Writing of the manuscript: LL, LG, NC, AD, and HP.

## CONFLICT OF INTEREST

The authors declare no conflict of interest.

## Supporting information


**Figure S1.** Fertility analysis of *teb‐5/rtel1‐1*.
**Figure S2.** Backcross experiments of teb‐5/rtel1‐1.
**Figure S3.** Mitotic anaphases in rtel1‐1/ku70‐1.
**Table S1.** List of used oligonucleotides for genotyping.
**Table S2.** List of primer combinations used for genotyping.

## Data Availability

The datasets supporting this study are available from the corresponding author upon reasonable request.
