# Supplementary material for: The DNA helicase RTEL1 is involved in the repair of replicative DNA damage independently of the alternative end joining and the DNA–protein cross‐link repair pathways in Arabidopsis
Source: Plant J. 2026 May 5;126(3):e70903. doi: 10.1111/tpj.70903 (PMC13143409; doi:10.1111/tpj.70903)
Supplement: Supplementary file 1 — Figure S1. Fertility analysis of teb‐5/rtel1‐1. Figure S2. Backcross experiments of teb‐5/rtel1‐1. Figure S3. Mitotic anaphases in rtel1‐1/ku70‐1. Table S1. List of used oligonucleotides for genotyping. Table S2. List of primer combinations used for genotyping. [file TPJ-126-0-s001.docx]

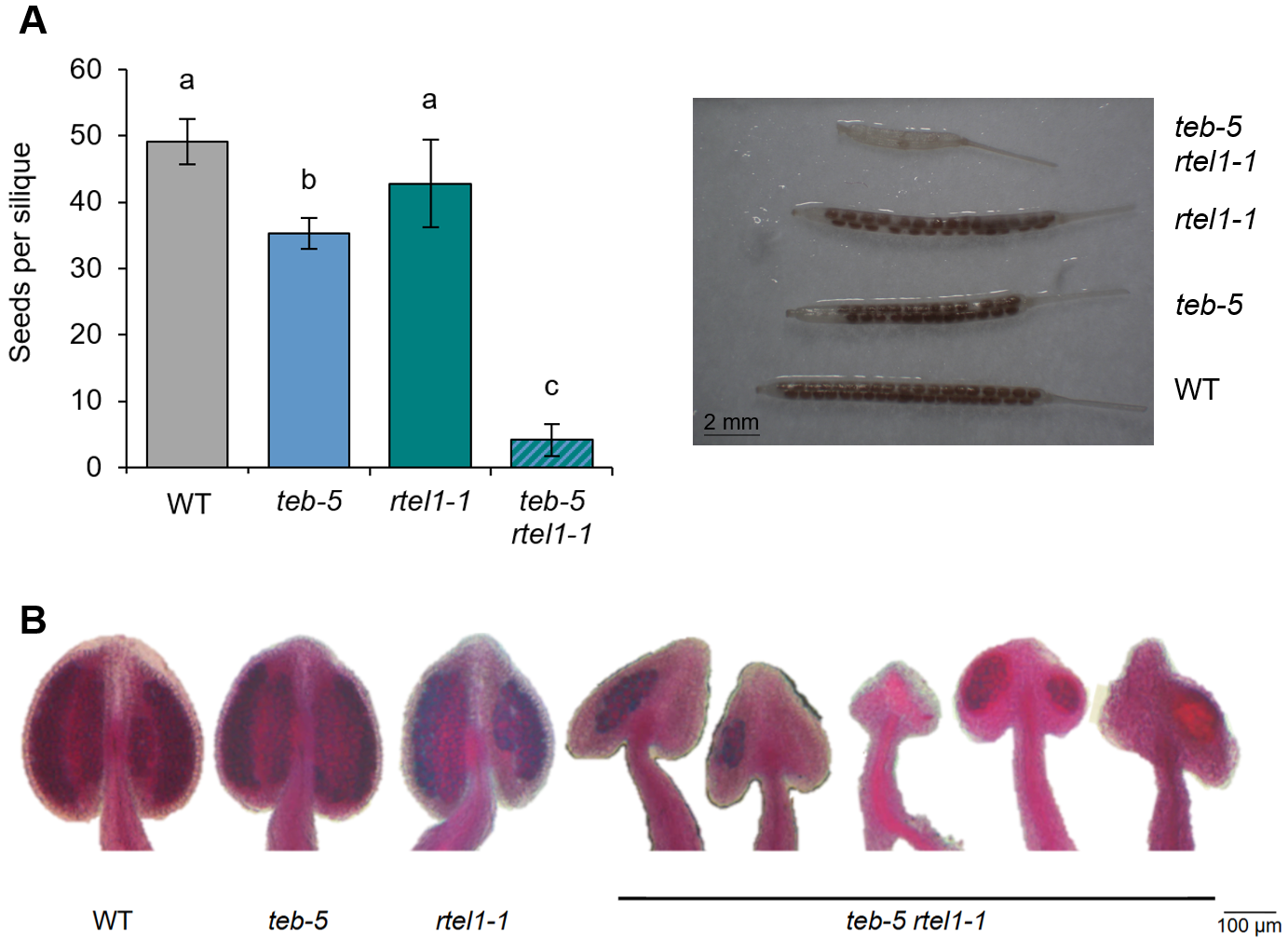
**Supplemental Figure 1: Fertility analysis of *teb-5/rtel1‑1***

**(A)** Quantification of the average number of seeds per silique showed a significantly reduced fertility of *teb-5/rtel1‑1* compared with the wild type and the respective single mutants (n = 5). Statistical differences were calculated using a one-way ANOVA followed by Tukey’s post hoc test and are presented as a ≠ b when p < 0.05.

**(B)** Alexander staining was used to analyze pollen viability. No dead pollen were detected in any of the examined lines but anthers of *teb-5/rtel1‑1* were severely deformed.


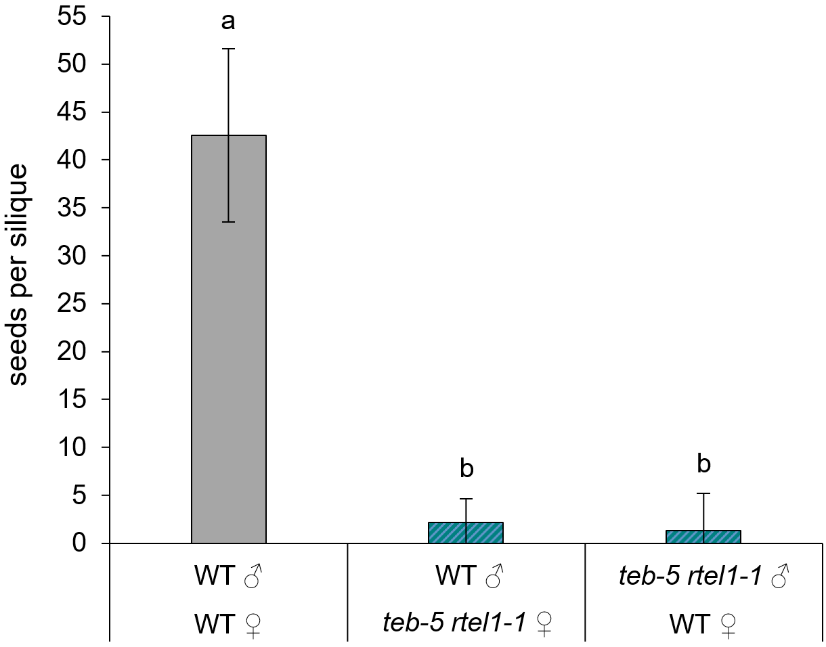


**Supplemental Figure 2: Backcross experiments of *teb-5/rtel1‑1***

Backcross experiments revealed that wild‑type (WT) plants crossed with *teb-5/rtel1‑1* produced significantly fewer seeds per silique than two wild‑type plants crossed with each other. Both crossing directions with the mutant exhibited comparable fertility (WT ♂ x WT ♀︎, n = 40; WT ♂ x *teb-5/rtel1‑1 ♀︎,*n = 31; *teb-5/rtel1‑1 ♂* x WT ♀︎, n = 40). Statistical differences were calculated using a one-way ANOVA followed by Tukey’s post hoc test and are presented as a ≠ b when p < 0.05.

**
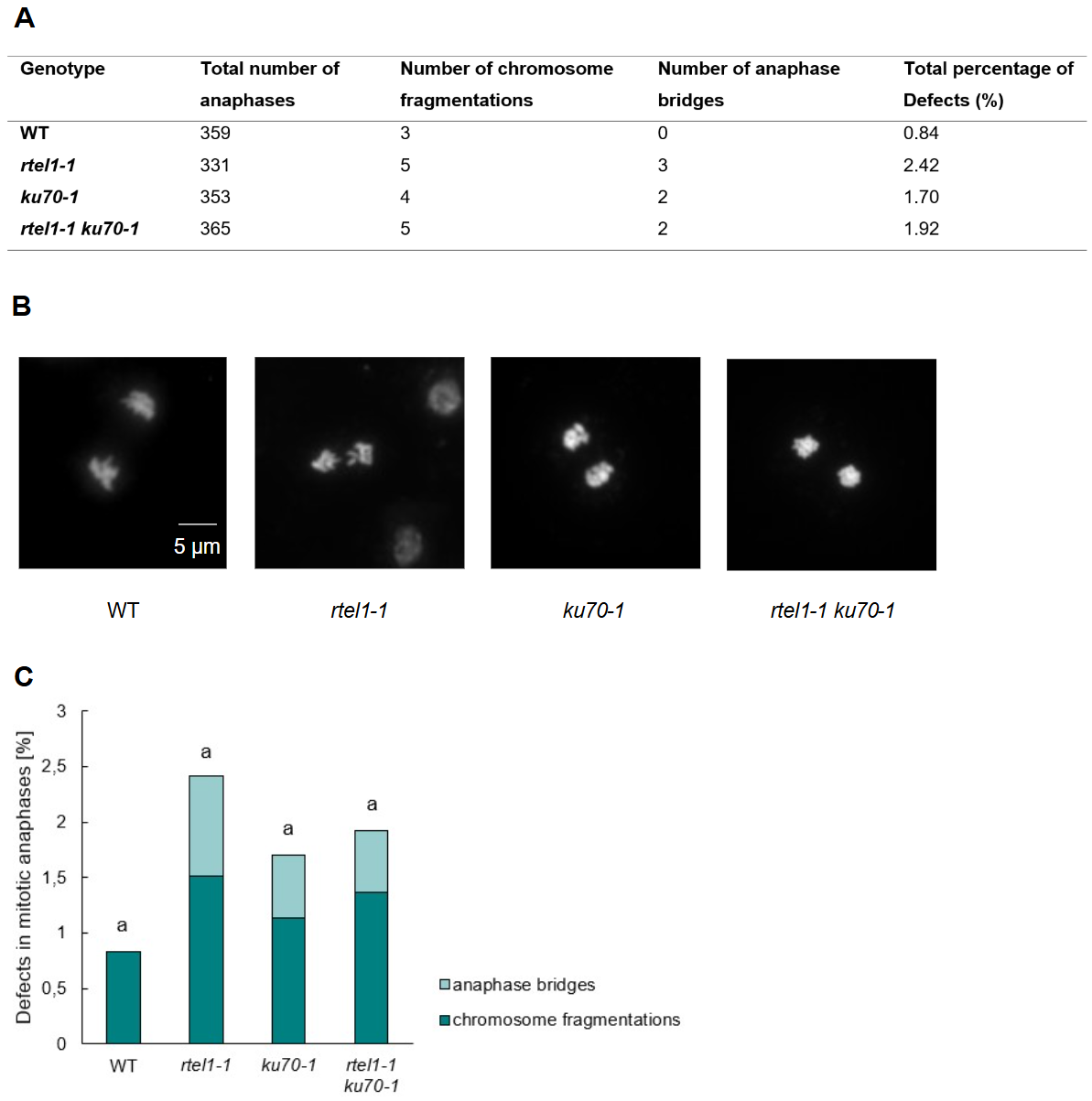
Supplemental Figure 3: Mitotic anaphases in *rtel1-1/ku70‑1*.**

**(A)** Shown is the quantification of mitotic anaphase defects, including chromosome fragmentations and anaphase bridges, in *rtel1‑1/ku70‑1* relative to single mutants and the wild type (WT).

**(B, C)** DAPI-stained chromosome spreads were analyzed to identify defects in mitotic anaphases. For each genotype, at least 300 mitotic anaphases derived from a minimum of three independent plants were analyzed. The *rtel1‑1/ku70‑1* double mutant exhibited similar frequencies of anaphase bridges and chromosome fragmentations compared with the wild‑type and the corresponding single mutants. Statistical differences were calculated using a two‑tailed Fisher’s exact test and are presented as a ≠ b when p < 0.05.

**Supplemental table 1: Sequences of the used oligonucleotides.**

| Primer name | Sequence (5’-3’) |
| --- | --- |
| ku70_fw2 | TTACTTTGTTGTTTCGGGTGC |
| ku70_rv2 | CTCTTGGCAAGTACACGCTTC |
| LBb1.3 | ATTTTGCCGATTTCGGAA |
| LBd1 | TCGGAACCACAATCAAACAG |
| RTEL1-IN5-fw1 | GGGTTACCAAACGATTATAC |
| RTEL1-IN7-rev1 | CGACACAGAATATAAAGAACA |
| teb-5 LP | ATTCATTGCTCGGCATCTATG |
| teb-5 RP | CGATTCATTGGATTGTTTTGG |
| WSS1A FW2 | GGCTTAGGAGGGTAAACCATGA |
| WSS1ARV1 | GCTATCACCACCAAGACGTTG |
| WSS1A-52 FW5 | GAGGCGTGCAAGTGAAATTC |
| WSS1A RV2 | CTGCTATCACCACCAAGACG |

**Supplemental Table 2: Primer combinations used for genotyping.**

| Genotype |  | Primer combination | Annealing temperature |
| --- | --- | --- | --- |
| *ku70-1* | WT DNA | ku70_fw2 / ku70_rv2 | 60 °C |
|  | T-DNA | ku70_rv2 / LBb1.3 | 60 °C |
| *rtel1-1* | WT DNA | RTEL1-IN5-fw1 / RTEL1-IN7-rev1 | 56 °C |
|  | T-DNA | RTEL1-IN5-fw1 / LBd1 | 56 °C |
| *teb-5* | WT DNA | teb-5 RP / teb-5 LP | 56 °C |
|  | T-DNA | teb-5 RP / LBb1.3 | 53 °C |
| *wss1A-3* | WT DNA | WSS1A FW2 / WSS1ARV1 | 56 °C |
|  | T-DNA | WSS1A-52 FW5/ WSS1A RV2 | 56 °C |
